# Editorial

**Published:** 1874-10

**Authors:** 


### Editorial.

---

AGAIN WE PRESENT OUR READERS with an unusually attractive *carte* of original matter, which crowds out our editorial lucubrations and pencilings. Of this we are far from complaining, whilst our readers will, we doubt not, accept the situation with inward satisfaction. If our subscribers shall but enjoy the perusal of our printed pages with something of the satisfaction which the manuscript has afforded us, we shall be more than content.

---

WE HAVE RECEIVED from the office of Dr. John M. Woodworth, Supervising Surgeon of the U. S. Hospital Service, a circular, soliciting information touching the *cholera epidemic* of 1873, to enable him to make a report upon the subject to the Congress of the United States. Any of our readers prepared to furnish to Dr. Woodworth the desired information can obtain his circular explanatory of what is wanted, by application to the Editor, or directly to Dr. W., at the Treasury Department, Washington, D. C. We trust that the profession will heartily respond to this call for information for the public welfare.

Dr. Woodworth has also favored us with a copy of the circular of September 8th, 1874, Treasury Department, touching the "Duties of U. S. Officers with reference to Quarantine and the Public Health."

A HINT IN GIVING IODIDE OF POTASSIUM.—Sir James Paget was the first to call the attention of the medical profession to the following interesting fact—namely, that carbonate of ammonia greatly increases the therapeutic action of iodide of potassium. I have had extensive experience in the treatment of syphilis, and have tried it, with the best results, and find that five grains of iodide of potassium, combined with three grains of carbonate of ammonia, are equal to eight grains of the potassium salt administered in the ordinary way.—*Cincinnati Lancet and Observer.*

